# Lithium Insertion into Graphitic Carbon Observed via Operando Kerr-Gated Raman Spectroscopy Enables High State of Charge Diagnostics

**DOI:** 10.1021/acsenergylett.2c01120

**Published:** 2022-07-18

**Authors:** Alex R. Neale, David C. Milan, Filipe Braga, Igor V. Sazanovich, Laurence J. Hardwick

**Affiliations:** † Stephenson Institute for Renewable Energy, Department of Chemistry, University of Liverpool, Peach Street, Liverpool L69 7ZF, United Kingdom; ‡ Central Laser Facility, Research Complex at Harwell, STFC Rutherford Appleton Laboratory, Harwell Campus, Didcot, Oxfordshire OX11 0QX, United Kingdom; § The Faraday Institution, Quad One, Harwell Campus, Didcot OX11 0RA, United Kingdom

## Abstract

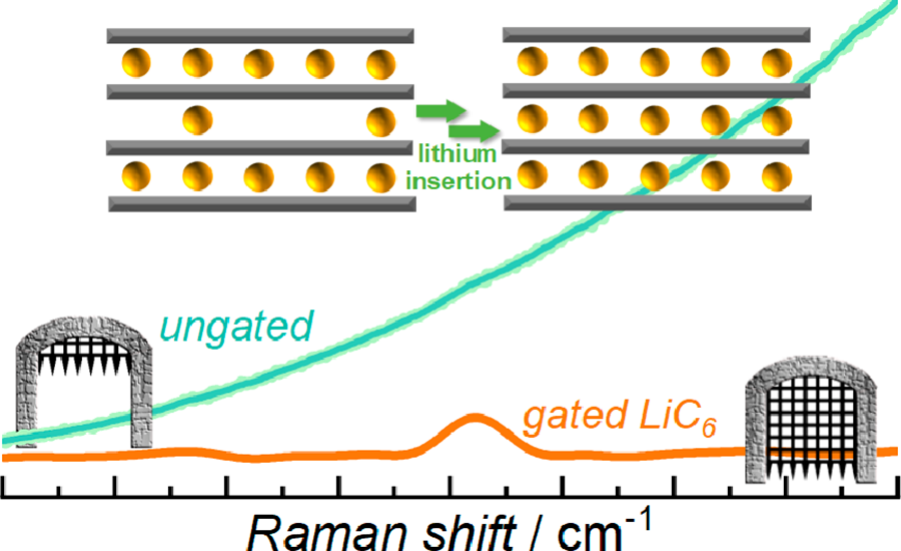

Monitoring the precise lithium inventory of the graphitic carbon electrode within the Li-ion battery, in order to assess cell aging, has remained challenging. Herein, operando electrochemical Kerr-gated Raman spectroscopy measurements on microcrystalline graphite during complete lithium insertion and extraction are reported and compared to conventional continuous-wave Raman microscopy. Suppression of the fluorescence emission signals via use of the Kerr gate enabled the measurement of the Raman graphitic bands of highly lithiated graphite where 0.5 ≤ *x* ≤ 1 for Li_*x*_C_6_. The broad graphitic band initially centered at ca. 1590 cm^–1^ for Li_0.5_C_6_ linearly shifted to ca. 1564 cm^–1^ with further lithiation to LiC_6_, thus offering a sensitive diagnostic tool to interrogate high states of charge of graphitic carbon-based negative electrodes.

Accurate diagnosis of the state of charge of electrodes is essential for improving lithium-ion cell lifetimes.^1,2^ In particular, practical techniques to rapidly and regularly monitor and distinguish between highly lithiated (charged) states of graphitic carbon over many cycles are essential to assign key Li-ion cell degradation processes operando.^3−5^ Nuclear magnetic resonance and X-ray and neutron diffraction methods have been shown to distinguish between stage 2 and stage 1 graphite intercalation compounds (GICs) under electrochemical/potential control; however, each method has its own particular limitations for routine analysis.^5−11^ Raman microscopy has been consistently described as a robust and accessible operando technique to identify the initial states of lithiation from dilute stage 1 through to stage 4 and stage 3. Beyond stage 2, however, background fluorescence and reduction of the optical skin depth result in spectra with broad features that make it challenging to obtain a reliable fit.^12−15^ As such, unlike for the earlier stages, numerical fitting of shifting Raman modes at high states of lithiation in stage 2 and stage 1 Li-based GICs cannot be achieved. Therein, in terms of assessing practical Li-ion cells, as the lithium inventory of the overall Li-ion cell reduces with continued cycling, this will result in incomplete lithiation of the graphite at high states of charge. As such, being able to monitor changes at high lithiation states in the low stages of GICs is vital to accurately diagnose cell failure mechanisms.

During the electrochemical cycling of Li-ion batteries with conventional Li[PF_6_]/carbonate-based electrolytes, fluorescent species are formed by parasitic decomposition reactions, primarily due to the instability of the non-aqueous electrolytes and side reactions that occur at the electrode surface. The increase in the background fluorescence emission arising from these components makes it more challenging to analyze Raman spectroscopic data on these cycled materials due to the spectroscopic overlap of Raman scattering and fluorescence emission.^16^ One methodology that can overcome the challenges of Raman/emission signal overlap is Kerr-gated Raman spectroscopy.^17,18^ It has previously been demonstrated that Kerr-gated Raman spectroscopy can be exploited to effectively remove the fluorescence background on uncycled, cycled, and aged battery and electrolyte materials and reveal the Raman scattering signals.^19^ Kerr-gated Raman spectroscopy is a technique that relies on exploiting the varied time domains of Raman scattering (fs–ps) and emission signals (ps–ns) following excitation by ultra-short laser pulses.

In this work, the use of operando Kerr-gated Raman spectroscopy is reported for the investigation of structural changes of intercalation in a Li-ion graphitic negative electrode using a conventional Li[PF_6_]/organic carbonate-based electrolyte. With a dedicated cell development to facilitate this operando methodology, the conventional and well-understood structural stage changes can be observed in both Kerr-gated Raman spectroscopic and electrochemical results. However, due to the efficacy of the Kerr gate in filtering out sufficient fluorescent signals, the results reported here retain some observable Raman spectroscopic information, even at low-stage GIC phases (i.e., high states of lithiation), with much greater clarity than has been achieved by conventional Raman microscopy techniques. Consequently, clear trends in the spectroscopic responses can be assigned as a function of the state of charge (lithiation), and a numerical fit of the changes occurring through the transition from stage 2 to stage 1 regions is obtained here. This creates a powerful tool for assessing high states of lithiation of the graphitic negative electrode.

Herein, an operando Li|graphite half-cell was assembled to facilitate the observation of structural changes by Kerr-gated Raman spectroscopy during electrochemical (de)intercalation with a free-standing synthetic microcrystalline graphite electrode. A Li metal disc was utilized as the counter electrode, and a 1 mol dm^–3^ solution of Li[PF_6_] in ethylene carbonate (EC) and dimethyl carbonate (DMC) (1:1 vol/vol) constituted the electrolyte formulation. The operando cell, modified specifically for the Kerr-gated Raman system used in this work, contained a viewport with a CaF_2_ window, through which the graphite working electrode can be exposed to the Raman excitation laser (Figure S1, described in further depth in the Supporting Information). The ultra-fast pulsed laser (400 nm) was raster scanned across the electrode surface to minimize damage, and spectra were collected while under galvano-/potentiostatic control.

The initial Kerr-gated Raman spectrum of the graphitic carbon electrode wetted with the electrolyte, at open circuit potential (OCP), is presented in Figure 1, along with the equivalent continuous-wave (CW) Raman spectrum and the Kerr-gated Raman spectrum of the bulk electrolyte. Therein, the 0 and 2 ps delay times spectra are provided for the Kerr-gated Raman spectrum, demonstrating the differences in the arrival time at the gate for signals across the studied spectral range due to dispersion of light signals introducing delays. The spectra show the two primary bands for graphitic carbon, G and a broad 2D overtone, appearing at 1586 and 2780 cm^–1^, respectively. The G band arises from the E_2g2_ mode of the sp^2^ carbons’ vibration in rings. The Kerr-gated Raman signals are also compared with the CW Raman spectrum (633 nm excitation) in Figure 1. The relative shift in the 2D band position results from the dependence of this double-resonance process on the excitation energy. The shift of the 2D band maximum, ca. 110 cm^–1^, corresponds reasonably well (for 633 nm (1.96 eV) and 400 nm (3.1 eV) excitation energies) with the approximate linear dispersion, 100 cm^–1^ eV^–1^, described in the literature.^20,21^ The D band observed in the CW Raman spectrum would also be expected to shift linearly by 50 cm^–1^ eV^–1^, equating to a difference of ca. 56 cm^–1^ for the 400 and 633 nm excitation lasers used here. This may account for the very minor rise in baseline intensity at ca. 1385 cm^–1^ in the Kerr-gated Raman spectrum, but the intensity is too low to be clearly resolved from the baseline noise.

**Figure 1:**
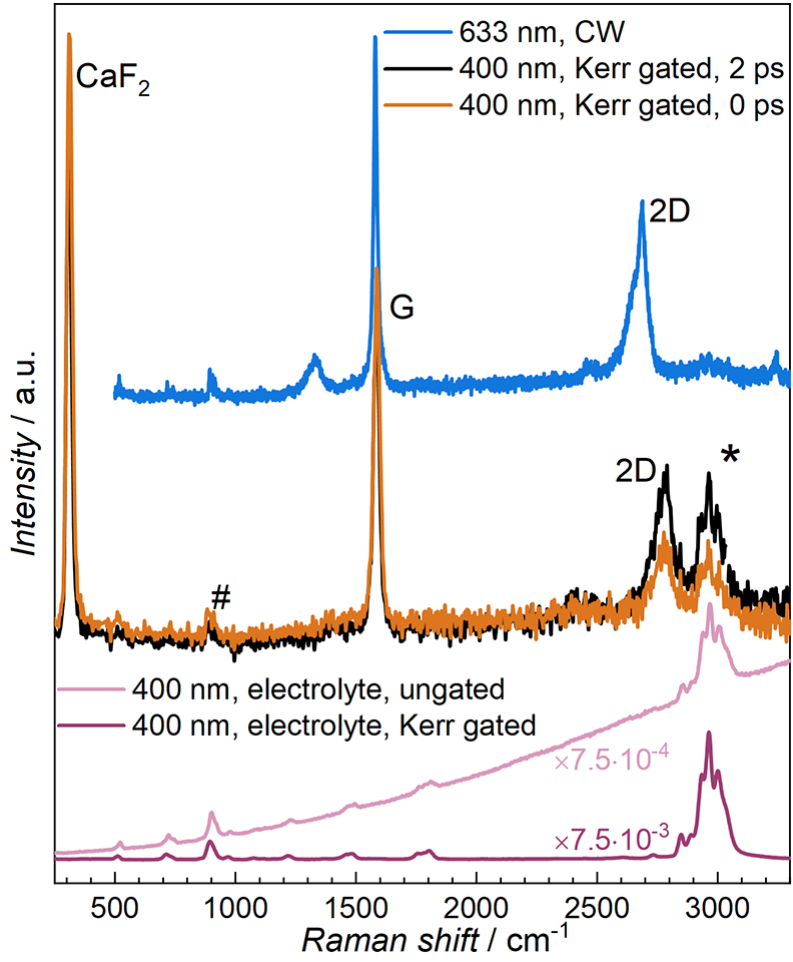
Raman spectra of the wetted graphitic carbon electrode and of the neat electrolyte using continuous-wave (633 nm) and Kerr-gated (400 nm) Raman. The graphite spectra show the characteristic G and 2D bands as well as primary electrolyte bands (*, #). The sharp band for the CaF_2_ window of the operando cell is also observed. The graphite spectra are normalized to the intensity of the G band, while the electrolyte signal intensities are significantly reduced to the scale of graphite spectra by the factors shown on each trace. The electrolyte spectra were reproduced from previous work.^19^

The largest intensity band at ca. 320 cm^–1^ corresponds to the CaF_2_ window of the electrochemical cell. The primary Raman scattering signals originating from C–H stretching modes of the EC/DMC electrolyte solvents within the wetted electrode are also seen in the broad peak centered around 2980 cm^–1^ (*). A less intense electrolyte signal centered around 900 cm^–1^ (#) is also observed and can be attributed to the ring-breathing and O–C–O bending modes of EC and DMC, respectively. The 400 nm Raman spectra of the bulk electrolyte collected with and without the Kerr gating are also provided in Figure 1, showing excellent agreement with the primary electrolyte bands observed in the wetted electrode. In previous work, the significant benefit of the Kerr gating on removing fluorescence/emission arising from this electrolyte formulation was introduced and demonstrated.^19^ In the absence of microscopic focusing on individual graphite particles that is routine with conventional Raman microscopes, spatially excluding or minimizing electrolyte bands (as well as the CaF_2_ window material) is challenging. This is evidenced by the difference in ratios between electrolyte and graphite bands in the CW and Kerr-gated spectra of the wetted electrodes, wherein the electrolyte bands are significantly reduced when collected with coupled microscopy. Consequently, for the Kerr-gated Raman technique, as highlighted by the massive emission baseline intensity of the ungated electrolyte spectra, pairing the cell design with the Kerr gating effect becomes more important here.

The operando Li|graphite half-cell was first discharged (graphite lithiation) at ca. C/7 (where 1C = 372 mA g^–1^ based on the theoretical capacity of graphite) from OCP (ca. 3.1 V vs Li^+^/Li) to a voltage limit of 10 mV. The cell was then held at a constant voltage 10 mV vs Li^+^/Li until the current decayed to <C/40. The cell was left overnight, allowed to relax, and charged (delithiation) the following day at C/5 to an upper voltage limit of 1.2 V vs Li^+^/Li. Kerr-gated Raman spectra were collected with 0 and 2 ps delay times while the cell was under galvanostatic and, subsequently, potentiostatic control to track spectral changes as a function of the electrochemistry. The discharge/charge voltage profile for the operando cell is presented in Figure 2a,b, displaying the conventional plateaus associated with structural stages of electrochemical intercalation of Li^+^ into the graphite layers of the electrode.^13,22−24^ Therein, the “stages” of GICs, defined by the stage index, refer to the number of graphene layers separating the intercalant layers. For example, stage 1 is the fully intercalated LiC_6_ where each graphene layer is adjacent to a Li intercalant layer, while in the early stages of charging, the GIC moves through stage 4 and stage 3, wherein four and three graphene layers, respectively, separate the intercalant layers. Within the electrochemical discharge of the cell (Figure 2a), the total charge passed on the first intercalation exceeds the theoretical capacity for forming LiC_6_ (i.e., where *x*(Li) = 1 in Li_*x*_C_6_ = 372 mAh g^–1^), due to established additional and irreversible reductive decomposition reactions that contribute to formation of solid electrolyte interphase (SEI) films on graphite electrodes. Based on the voltage profiles and total discharge capacity, the excess capacity attributed primarily to SEI formation is estimated at ca. 128 mAh g^–1^, or *x*(Li) ≈ 0.35.

**Figure 2:**
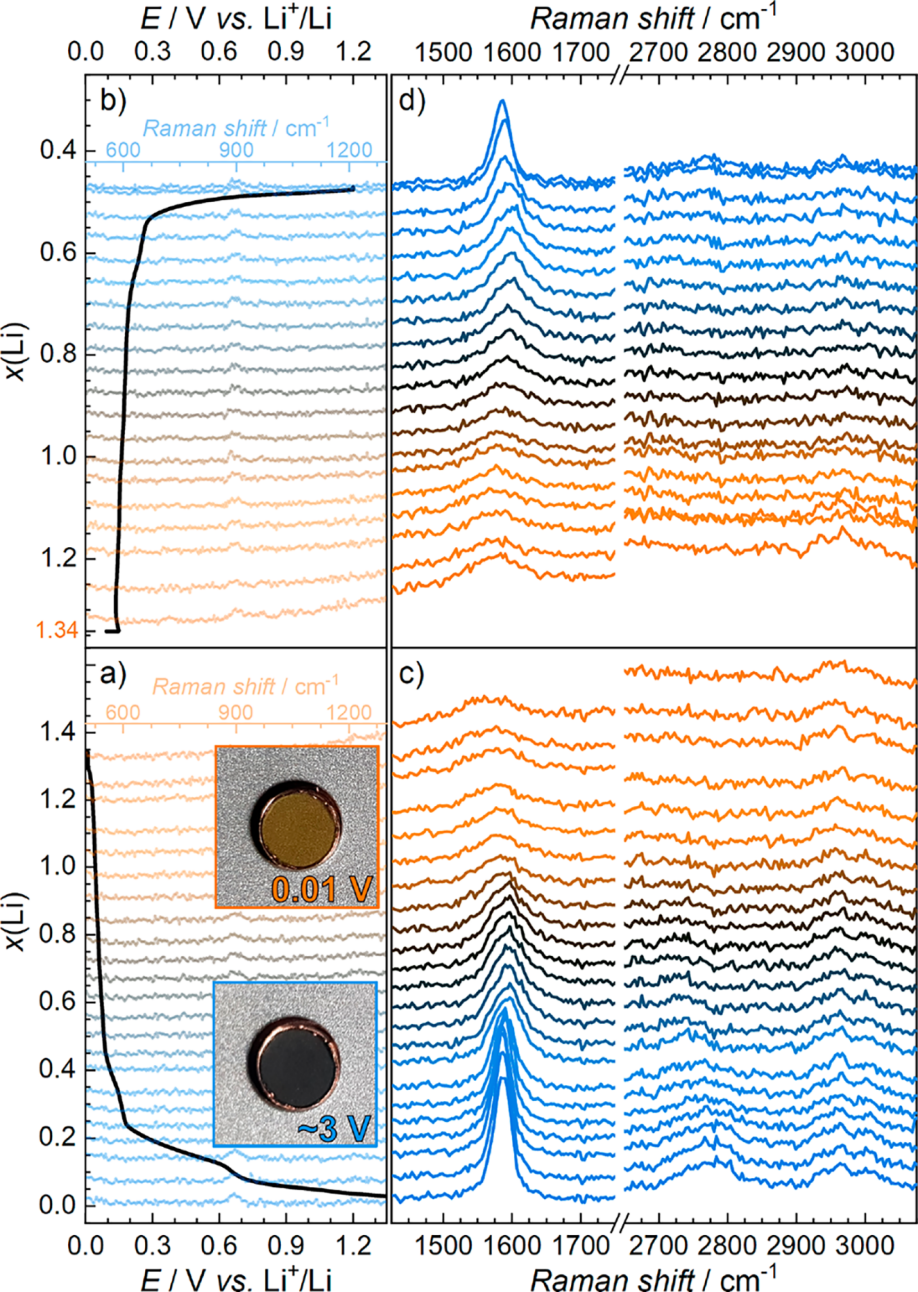
(a, b) Voltage profiles of the graphite electrode and (c, d) the operando Kerr-gated Raman spectra collected at 2 ps delay times showing the primary G and 2D graphite bands at 1586 and 2780 cm^–1^, respectively (electrolyte bands at ca. 2980 cm^–1^). Spectra are stacked as a function of the depth of lithiation (*x*(Li)). Insets in (a) show images of the electrode via the optical window before and after full lithiation to LiC_6_. The faded spectra in (a) and (b) show the low-wavenumber (500–1300 cm^–1^) spectral regions to highlight the intersection with the depth of lithiation (accounting for the large emission baselines at higher wavenumbers for high depths of lithiation).

Selected Kerr-gated Raman spectra collected under galvano-/potentiostatic control at the 2 ps delay time are provided without baseline subtraction in Figure 2c,d. Therein, spectra are stack-plotted as a function of the depths of (de)lithiation in accordance with the voltage profiles shown in Figure 2a,b. The equivalent spectra for the 0 ps delay time, wherein the high wavenumber regions are slightly enhanced, are provided in Figure S2 in the Supporting Information. The optical images provided in the insets of Figure 2a show the color change after the discharge step from black pristine graphite to dark gold across the full electrode surface, attributed to the formation of fully intercalated LiC_6_.^23^

During the lithiation step, the two prominent graphite bands were observed to change as a function of the cycling: the G band at 1586 cm^–1^ and the broad 2D band centered at ca. 2780 cm^–1^. Concurrently, the magnitude of the baseline arising from emission signals grows as a function of the lithiation step (observed more prominently in the 0 ps delay time spectra in Figure S2). This effect of increasing emission baseline, as well as the primary changes in the observed graphite bands, is mostly reversed during the delithiation step. Considering the lithiation step, beginning at ca. 0.5 V vs Li^+^/Li, the G band first undergoes a gradual blue-shift from ca. 1585 cm^–1^ to ca. 1595 cm^–1^ at 0.15 V vs Li^+^/Li, corresponding to approximately −29 cm^–1^ V^–1^. This is in good agreement with previous measurements using CW Raman microscopy with the same graphitic carbon material^14^ and work done with other graphite materials.^25^ At this point in the lithiation step, the G-band feature begins to significantly broaden and subsequently continuously red-shift for the remaining process. The broadening observed here (from 0.2 V vs Li^+^/Li to ca. 0.06 V vs Li^+^/Li) is related to a splitting of the G band into two distinct modes associated with the formation of stage 3 and stage 4 intercalation phases in the material. When observable, these features are attributed to the interior E_2g2_(i) (ca. 1575 cm^–1^) and bounding E_2g2_(b) (ca. 1600 cm^–1^) modes, where the interior modes are from layers non-adjacent to the Li^+^-intercalated layer planes (and the bounding modes are adjacent).^12−14,26^ Within the measured Kerr-gated spectra, these E_2g2_ features cannot be distinguished and are observed only as a broad single feature (discussed later). As the intercalation proceeds toward high depths of lithiation toward stages 2 and 1, the interior mode is lost (since no non-adjacent graphene layers remain), and this has been confirmed by CW Raman microscopy experiments.^12,14,27^ At this point, where the cell voltage is <60 mV vs Li^+^/Li, the remaining G-band-related features in the Kerr-gated Raman spectra red-shift continuously until the lithiation step is completed. These final transitions through stage 2 and stage 1 of the intercalation process, as will be explored in greater depth later, have been difficult to reliably follow using conventional Raman spectroscopic techniques, and the efficacy of the Kerr-gated Raman method in removing the emission baseline plays a key role in enabling this analysis.

The changes in the broad 2D band centered around 2770 cm^–1^ upon lithiation primarily relate to a large red-shift and loss of Raman scattering intensity. These changes begin after the cell voltage drops below 0.7–0.6 V vs Li^+^/Li. Therein, a small red-shift in the peak maximum downward by ca. 15 cm^–1^ occurs from 0.6 to 0.2 V vs Li^+^/Li. Thereafter, the 2D band feature red-shifts by a further 45–50 cm^–1^ toward a minor broad peak centered at 2710 cm^–1^ in the spectra collected at a cell potential of ca. 60–70 mV vs Li^+^/Li (where *x*(Li) ≈ 0.65). These changes can be most clearly tracked by utilizing the 0 ps delay time spectra, where the intensities in the higher wavenumber regions are greater (see Figure S2). This trend is congruent with previous observations by conventional CW Raman microscopy.^14,26^ At greater states of lithiation covering the transitions through stages 2 and 1, it is postulated that the 2D band becomes no longer observable since all the graphene layers become charged.^28^ However, as can be seen more clearly in baseline-subtracted spectra in the operando Kerr-gated Raman cell provided in Figure S3, a small, and continuously red-shifting (as low as 2665 cm^–1^), broad feature could still be observed above the zero-intensity baseline at greater depths of lithiation. Given the low signal intensity and the continued growth of the competing emission signals therein, this signal becomes more difficult to reliably distinguish from the spectral noise but can be reliably observed in all but the final spectra (i.e., at *x*(Li) = 1.34). Overall, due to the efficacy of fluorescence suppression by exploiting the Kerr gate, both G and 2D features are still visible beyond stage 2 and through the transition to stage 1 intercalation compounds. Previous explanations cited the decrease in optical skin depth as the GIC became more conductive.^12−15^ Though this rationale can still be invoked to explain the weakening of the Raman signal, it would not be expected to lead to a complete loss of signal.

During the delithiation step (Figure 2d and Figure S2d), the general sequence of observations described above for the observed G-band feature occurs in reverse. Therein, a slightly higher current was employed, and, as such, the cell overpotentials and delays in lithium diffusion throughout the graphitic electrode are likely to impose small errors in the true cell potential and state of lithiation assignments for each spectrum. Furthermore, a difference can be observed between the last spectra collected on lithiation and the first spectra collected on delithiation (particularly in the G-band region). Therein, between the two steps, the cell had to be left to relax at open circuit overnight. The Raman spectrum of the lithiated graphite electrode was then measured without the Kerr gate (discussed later) before beginning the delithiation step. Removing and then re-implementing the Kerr gate into the experimental setup required some changes to be made to the equipment, and the cell required repositioning and refocusing of the pulsed laser beam onto the working electrode surface. In addition to the relaxation of the graphite electrode, these factors could account for some of the differences in this spectral pair.

Application of the Kerr gating to measure Raman spectra using high-energy, ultra-fast pulse laser excitation clearly provides important benefits to systems that undergo significant fluorescence emission behavior, as highlighted in Figure 3. Subsequently, the Kerr-gated Raman technique is compared with more conventional CW Raman microscopy that we and other groups have reported for lithiation of graphite^12,14,25^ and other electrochemical systems previously.^29−31^ Therein, as for the CW Raman spectra shown in Figure 1, a 633 nm excitation laser was used here. One significant difference between the two methodologies relates to the coupled use of the microscope in CW Raman. As discussed previously, this allows careful focusing on individual electrode particles, which aids in the reduction of Raman (and emission) signals arising from the electrolyte. Conversely, the Kerr-gated Raman spectra are collected by rastering the laser spot across the electrode surface, which ensures the measured signals are averages of the studied surface. This may alleviate, or help to account for, local inhomogeneities in the states of lithiation across the electrode structure that otherwise require careful experimental and equipment design to overcome.^27^

**Figure 3:**
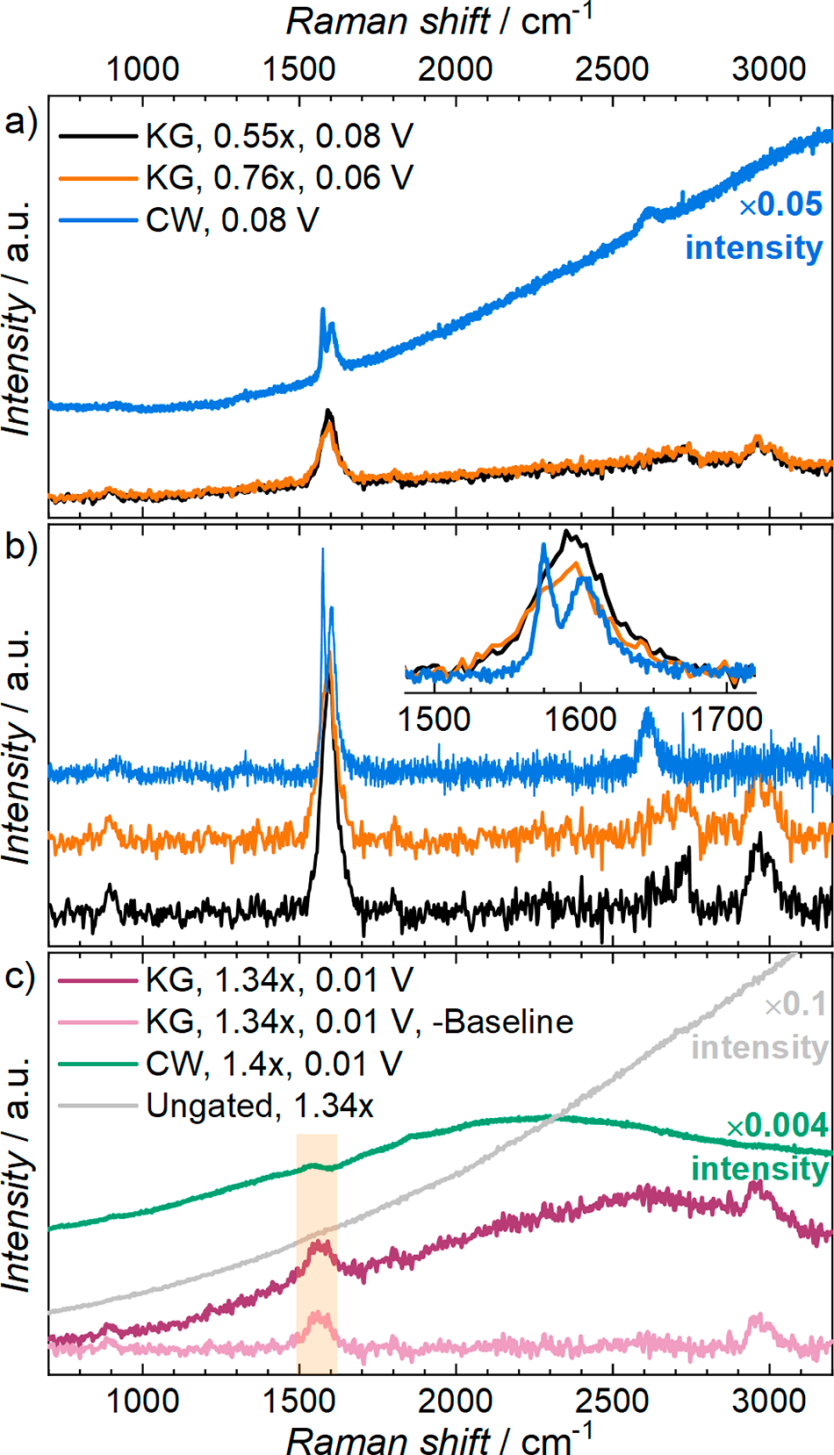
Comparison of Kerr-gated (KG, 400 nm excitation) and continuous wave (CW, 633 nm excitation) Raman spectra of the graphitic electrode (a, b) during and (c) at the end of the lithiation process. The ungated spectrum of LiC_6_ at 1.34*x* is also shown in panel c. Spectra in panel b are stacked following baseline subtraction of spectra in panel a, and the inset shows the observed difference in the G-band splitting. The cell voltage at the time of spectral collection and the corresponding degree of lithiation (i.e., *x* in Li_*x*_C_6_) are shown for each trace.

To explore the differences in the two methods, the intercalation of lithium into an equivalent free-standing graphite electrode was also probed using CW Raman microscopy (using a 633 nm excitation laser). The operando CW Raman and operando Kerr-gated Raman spectra of the two graphite electrodes at two depths of lithiation (*x*(Li) ≈ 0.6–0.8 and >1.3) are provided in Figure 3. At both lithiation states (Figure 3a,c), both methodologies measure a degree of emissions signals; however, the relative effect on the baseline shape and magnitude is significantly larger for the CW Raman spectra. Nevertheless, for the spectra collected earlier in the lithiation processes (Figure 3a,b), the key graphite bands can still be observed by both techniques. Critically, as highlighted by the inset panel in Figure 3b, the CW Raman spectrum allows clear observation of the splitting of the G-band to the distinct interior E_2g2_(i) and bounding E_2g2_(b) modes (discussed above). Within the Kerr-gated Raman spectra, this change manifests as an observed broadening of the G-band without resolving the splitting of the peak. This difference could arise through a combination of factors: the lower spectral resolution of the Kerr-gated Raman technique; different sensitivities of the interior modes to the 400 and 633 nm excitation energies of Kerr-gated and CW Raman methods, respectively; and the difference in homogeneity of the material under detection. Therein, the spectral resolution for the Kerr-gated Raman technique described here is estimated at 17 cm^–1^ or better, whereas the spectral resolution of the lab-based CW Raman microscopy would be better than 4 cm^–1^. Given that the peak-to-peak separation of the E_2g2_(i) and E_2g2_(b) modes in the CW Raman spectra in Figure 3b is >25 cm^–1^, this suggests that the spectral resolution of the Kerr-gated Raman technique is not the only limiting influence. Considering the latter factor of the homogeneity of the sample under investigation, when collecting the Kerr-gated Raman spectra, the raster scanning pattern of the sample ensures an average measurement across ca. 25 mm^2^ of the electrode, which could result in broadening of the resulting signals. Conversely, as described above, CW Raman microscopy enables accurate focusing and analysis of individual graphite flakes, probing a geometric area of <5 μm^2^, in which the inhomogeneity of the studied area is expected to be minimized. Such inhomogeneity in graphite electrodes during lithium intercalation has previously been demonstrated using operando/in situ CW Raman microscopy to compare spectra of different regions of the working electrode under the same cell voltage conditions.^13,27^

However, at greater states of lithiation toward pure LiC_6_ (Figure 3c, wherein the cell is held at a constant voltage of 10 mV vs Li^+^/Li, where *x*(Li) > 1.3), the CW Raman spectra tend to become overwhelmed. This is due to a combination of the growth in the overlapping emission signals that swamp the baseline and the reduction in Raman scattering intensity as the increased conductivity of the low-stage GICs reduces the optical skin depth of the probe beam. This makes it nearly impossible to identify any Raman peaks relating to the LiC_6_ electrode by CW Raman. As with CW Raman spectra, the changes in the observed bands in the Kerr-gated Raman spectra are also coupled with continuous growth in the emission baseline throughout the lithiation process. This is most prominent in the high wavenumber regions and contributes in part to the reduction of the 2D signal as lithiation proceeds below 60–70 mV vs Li^+^/Li (where *x*(Li) ≈ 0.75). However, while the growth in the observed baseline in the Kerr-gated Raman spectra does demonstrate that not all of the emission signals from the lithiated graphite working electrode are completely filtered by the Kerr gating effect, the comparative “ungated” spectrum presented in Figure 3c is featureless. This demonstrates that attempting to measure the electrode Raman signals (with 400 nm excitation pulses) at high depths of lithiation becomes impossible herein without the use of the Kerr gate. Conversely, the Kerr-gated Raman spectra retain clear peak features, even in the fully lithiated state of LiC_6_. This can be attributed to the effective suppression of the emission signals by the optical gate. As such, small bands relating to the graphite G-band (1565 cm^–1^) and the primary electrolyte bands centered at 2980 and 900 cm^–1^ are still observable. While the altered G-band feature aligns reasonably well with a small bump in the CW Raman spectra (highlighted at ca. 1550–1565 cm^–1^ in Figure 3c), such a feature would be impossible to confidently assign due to the magnitude and shape of the resulting emission baseline. Therein, the reduction in the absolute amount of emission detected during collection of Kerr-gated Raman spectra reveals these peaks clearly and, therefore, permits an unambiguous baseline subtraction, providing the most well-defined Raman spectrum for electrochemically formed LiC_6_ reported with Li[PF_6_]-based electrolytes.

The massive fluorescence/emission signal observed in highly/fully lithiated LiC_6_ by ungated ultra-fast laser-pulsed Raman and conventional CW Raman (Figure 3c) ensures that it becomes difficult (or impossible) to reliably probe the high states of lithiation (i.e., state charge of graphite and the lithium inventory as a negative electrode) by these methods. Thus, quantitative analysis of the state of charge for a Li-ion cell graphite electrode at high depths of lithiation is not achieved by conventional Raman spectroscopy. This can be attributed, in part, to the growing fluorescence/emission signals from decomposition products (most notable with the higher energy 400 nm pulsed laser excitation), as well as to the reduction in Raman scattering intensity of the GIC material as optical skin depth reduces as it moves from stage 2 to stage 1 of intercalation. Due to the different excitation wavelengths used for CW (633 nm) and Kerr-gated (and ungated) Raman (400 nm), the spectra of LiC_6_ reveal the expected differences in the shape and magnitude of the different competing emission baselines. While the 400 nm excitation would be expected to result in greater levels of emission (as supported by the ungated spectra in Figure 3c), the comparisons of spectra presented in Figure 3c clearly show the benefits of exploiting the Kerr gating effect for studying these materials. Therein, the ability of the Kerr gate to filter out a sufficient portion of the overlapping emission signals from the detected Raman spectra reveals a clear, detectable G-band feature even at the end of the lithiation step in *pure* LiC_6_ (Figure 3c). Furthermore, crucial shifts to lower wavenumbers and a broadening in the observed G-band feature are observed as lithiation of the graphite electrode proceeds to the later stages (see Figure 2c). The ability to observe this trend, and to reliably process the collected spectra, creates the unique opportunity to track the changing Raman modes and, thus, helps to assign the state of charge as the highly/fully lithiated electrode moves through stage 2 to stage 1 intercalated graphite.

Therein, the remaining observed G-band features were fitted by a single Lorentzian peak in the spectra collected below 60 mV, where the measured depth of lithiation exceeded *x*(Li) = 0.75. As discussed above, by estimating the charge contributions to irreversible SEI formations as ca. 128 mAh g^–1^ or *x*(Li) = 0.35, the range of states of lithiation of *x* in Li_*x*_C_6_ studied herein is, therefore, ca. 0.45 < *x* < 1. Moreover, the details of the peak fitting at the different states of lithiation are provided in Table S1, and the trends in the peak positions are presented as functions of the state of lithiation and the cell voltage in Figure 4. A linear approximation of both data sets (from *x*(Li)-SEI = 0.48 to 1) is provided in Figure S4. Therein, as the graphite intercalation proceeds through stage 2 and stage 1, the G-band signal shifts by approximately −4.5 ± 0.3 cm^–1^ (0.1·Li)^−1^. With respect to the cell voltage, this observed shift approximates at −0.53 ± 0.3 cm^–1^ mV^–1^. Furthermore, upon delithiation of the LiC_6_ electrode, the G-band signal increases back toward the original higher wavenumber region along a matching gradient with respect to *x*(Li) (see Figure S5).

**Figure 4:**
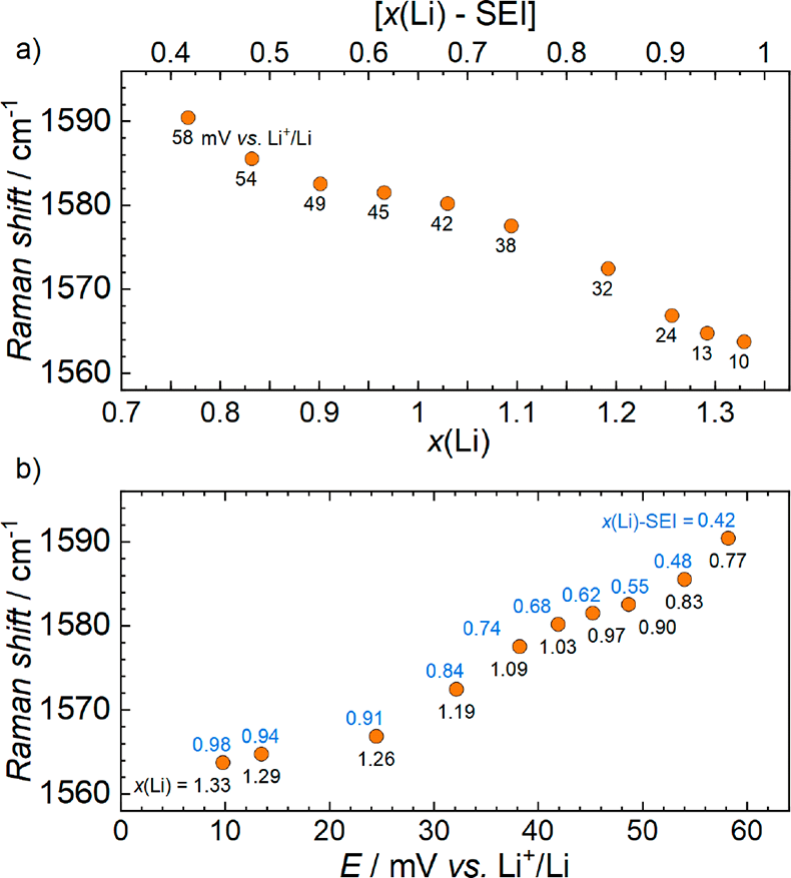
Peak positions of the fitted G-band peak at high depths of lithiation, plotted as functions of (a) the depth of lithiation (*x*(Li)) and (b) the cell voltage (*E*). The top *x*-axis of (a) represents an estimation of the state of lithiation in Li_*x*_C_6_ (where 0 < *x* < 1) by subtracting charge contributions from the irreversible SEI formation (estimated as 0.35*x*(Li)). The data labels in (a) show the associated cell voltage (in mV vs Li^+^/Li). The top (blue) and bottom (black) data labels in (b) show the associated state of lithiation with and without subtraction of the SEI contribution, respectively.

Therein, it is important to acknowledge that the peak positions will have an associated assignment error limited by the spectral resolution and relatively low Raman scattering signal intensity of the present Kerr-gated Raman technique on cycled graphite electrodes. Additionally, the nature of the cell configuration and the applied current densities may incur overpotential errors on the apparent cell voltage (making a direct relation between electrode voltage and depth of lithiation difficult). The weaker Raman scattering intensity of the present Kerr-gated Raman technique described here means it is difficult to identify any surface species generated during formation of the SEI on the graphite surface. Such observations may require improvements to the sensitivity of the equipment used and investigations into how to optimize the power, spot size, and bandwidth of the Raman probe laser to maximize signal collection. Furthermore, it is important to consider the exploitation of surface enhancements techniques, as has been reported on previously for CW Raman microscopy investigations of battery materials.^30,32^

However, the ability to maintain an observable (and lithium capacity dependent) graphite band at low stages of electrochemical intercalation of lithium into graphite, by effectively reducing the amount of detected emission signals, is a key strength of the Kerr-gated Raman technique demonstrated in this work. Therein, the observed trends demonstrate how Kerr-gated Raman spectroscopy opens the possibility to create an effective probe of the state of lithiation in highly charged graphite-based working electrodes. This would be an important tool in probing the remaining lithium inventory in *fully charged* graphite electrodes as they cycle in Li-ion full-cells/batteries. As well as seeking to develop the technique to improve sensitivity and resolution (spatial and spectral), demonstrating the spectroscopic trends observed here in highly lithiated graphite after cycling (several cycles up to hundreds of cycles) would be an important next stage in the development of the Kerr-gated Raman spectroscopic technique as an important diagnostic tool for Li-ion battery analysis.

In summary, Kerr-gated Raman spectroscopy has been shown to be an effective technique to investigate the intercalation behavior of electrode materials operando in high background emitting electrolytes, whereby operando Kerr-gated Raman spectroscopy followed the complete electrochemical lithiation and delithiation of a graphitic electrode. The significant reduction in fluorescence emission signals via use of the Kerr gate in operando allowed the measurement of the Raman band of highly lithiated graphite where 0.5 ≤ *x* ≤ 1 for Li_*x*_C_6_. This band had been challenging to observe due to the increasing emission background. The broad graphitic band initially centered at ca. 1590 cm^–1^ at Li_0.5_C_6_ shifted linearly to ca. 1564 cm^–1^ with further lithiation to LiC_6_. Therein, a numerical fit of the trend was obtained, thus providing a sensitive diagnostic tool to probe high states of charge for the graphite negative electrode in the Li-ion cell and to monitor aspects of cell degradation during cycling.

## Supporting Information Available

Operando Kerr-gated Raman spectra at the 0 ps delay time for the graphite working electrode in the Li|graphite half-cell; selected baseline-subtracted spectra at 0 and 2 ps delay times; peak-fitting information for the G-band feature at high states of lithiation; linear fitting of the fitted G-band Raman peak positions as functions of both the cell voltage and the depth of lithiation to LiC_6_ (PDF)
