# Supplementary material for: Lithium Insertion into Graphitic Carbon Observed via Operando Kerr-Gated Raman Spectroscopy Enables High State of Charge Diagnostics
Source: ACS Energy Lett. 2022 Jul 18;7(8):2611–8. doi: 10.1021/acsenergylett.2c01120 (PMC9380014; doi:10.1021/acsenergylett.2c01120)
Supplement: Supplementary file 1 — nz2c01120_si_001.pdf [file nz2c01120_si_001.pdf]

Supporting Information

Lithium Insertion into Graphitic Carbon Observed via Operando Kerr Gated Raman Spectroscopy Enables High State of Charge Diagnostics

*Alex R. Neale,[a,c] David C. Milan,[a,c] Filipe Braga,[a] Igor V. Sazanovich,[b] Laurence J. Hardwick[a,c]**

[a] Stephenson Institute for Renewable Energy, Department of Chemistry, University of Liverpool, Peach Street, Liverpool, L69 7ZF, United Kingdom.

[b] Central Laser Facility, Research Complex at Harwell, STFC Rutherford Appleton Laboratory, Harwell Campus, Didcot, Oxfordshire, OX11 0QX, United Kingdom.

[c] The Faraday Institution, Quad One, Harwell Campus, Didcot, OX11 0RA, United Kingdom.

Corresponding Author

hardwick@liverpool.ac.uk

Experimental methods

The operando Raman cell - To measure the Kerr gated Raman spectra of electrodes under electrical stimulation, a modified cell base and sample holder was designed to be coupled with the PEEK body of the ECC-Opto cell by EL-Cell and is illustrated in Figure S1. Therein, the baseplate was custom built for specific application in the Kerr gated Raman configuration, and a widened window opening (8 mm) was employed to accommodate the 5x5 mm square raster pattern of the excitation laser. A CaF_2 window (0.5 mm thick) allows the Raman laser to be focused on the graphite working electrode (WE) for the operando measurements. The Raman grade CaF_2 window (Crystran) shows only a single Raman peak centered at 320 cm^{-1} making this compound the best candidate for the cell window. The cell was hermitically sealed using O-rings under compression and the counter electrode (CE) current collector was spring-loaded to apply compression to the electrode stack under investigation. The sample holder, in which the sealed cell is placed, is connected to a motor that drives the sample through the raster scanning pattern during measurement to avoid laser damage to the sample.

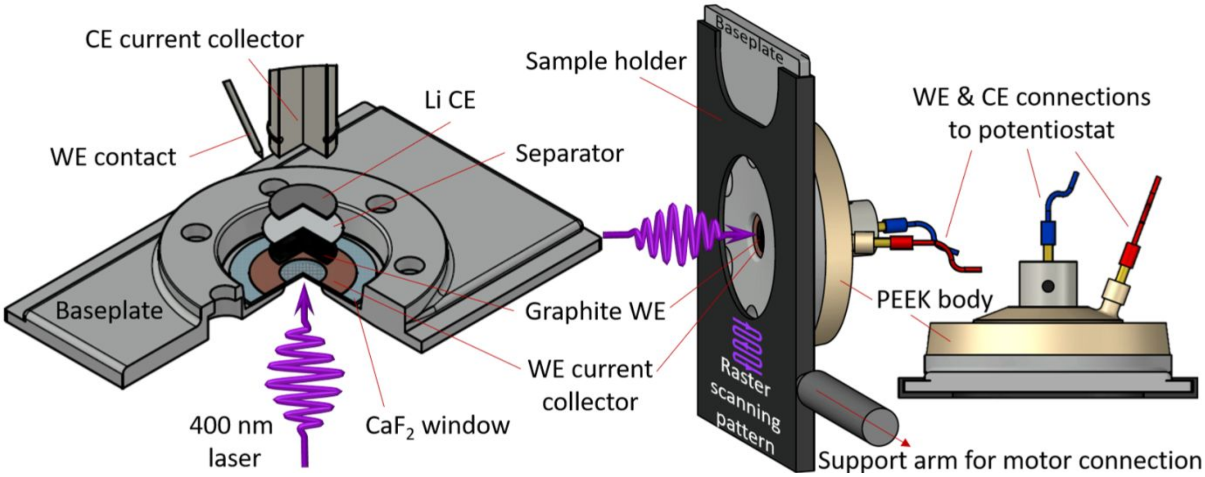

Figure S 1. Schematic of the operando Raman cell assembly showing the electrode stacking and contacts (left) and the cell within the Kerr gate Raman sample holder system (right) attached to the motor system (not shown) that provides the raster scanning motion during spectra collection.

The preparation of the free-standing graphite electrodes used here has been described previously[2]. As per prior work, synthetic microcrystalline graphite (SFG-6, Imerys, average particle size of 6 µm) was employed as the active material, with polyvinylidene fluoride binder and dibutyl phthalate as plasticizer. Following extraction of dibutyl phthalate from the films with

diethyl ether, the porous free-standing films punched to 10 mm disks and dried at 80 °C under vacuum inside the glovebox antechamber and taken directly into the Ar-filled glovebox (H_2O, O_2 ≤ 0.1 ppm).

The operando cell was prepared and sealed inside the Ar-filled glovebox as described in Figure S1 in a two-electrode configuration, before being transferred to the Kerr gated Raman lab. The counter electrode was a polished Li metal disk (10 mm diameter, 0.38 mm thick) pressed onto the stainless-steel current collector, and the separator (glass fiber, Whatman GF/F) was wetted with a conventional Li-ion battery electrolyte, 1 M $Li[PF_6]$ in ethylene carbonate/dimethyl carbonate (1:1 by vol). A copper foil ring (8 mm ID, 0.05 mm thick) was employed as the current collector for the free-standing graphite electrode. The electrochemical measurements were conducted simultaneously with spectroscopic measurements (below) using a SP-150 potentiostat/galvanostat (BioLogic Science Instruments). Therein, operando Li|graphite half-cell was firstly discharged (graphite lithiation) at *ca.* C/7 (where 1C = 372 mA g^{-1} based on the theoretical capacity of graphite) from OCP (*ca.* 3.1 V *vs.* Li^+/Li) to a voltage limit of 10 mV. The cell was then held at a constant voltage 10 mV *vs.* Li^+/Li until the current decayed to <C/40. The cell was left overnight and allowed to relax. The following day, before starting the charge (delithiation) step, the ungated Raman spectra of the LiC_6 working electrode was collected by removal of the CS_2 Kerr gated (described below) from the beam path. Subsequently, the Kerr gate was then reintroduced and, after refocusing of the excitation laser, the cell was charged at C/5 to an upper voltage limit of 1.2 V *vs.* Li^+/Li.

Operando Kerr gated Raman spectroscopy - Operando Kerr gated Raman measurements were conducted in conjunction with electrochemical measurements at the ULTRA laser facilities (Central Laser Facility, Rutherford Appleton Laboratories). *In lieu* of the coupled electrochemical measurements introduced in this work, the experimental details for the Kerr gated Raman measurements have been described in full in our previous work.[1] Therein, the Kerr gating system utilized a ps arm of the Thales dual-beam titanium sapphire laser that produced 0.8 mJ at a 10 kHz repetition rate.[2] The pulse duration was tuneable to between 1 and 3 ps. To achieve the optical

gating effect, carbon disulfide (CS_2) in a 2 mm cell was placed between two crossed polarizers and the Kerr effect was induced in the CS_2 medium with the 800 nm gating beam (0.5 mm spot) with a 45° polarization with respect to the polarizers. The Raman signal of the working electrode sample was probed using a 400 nm Raman probe beam with ps pulse lengths. The 400 nm Raman probe beam was produced by splitting some of the 800 nm beam with an optical beamsplitter, and sent into a frequency doubling β-barium borate crystal. The Raman probe beam spot size on the working electrode sample was set to a 150 μm. The Raman signal was detected at parallel polarization. A computer-controlled motorized linear stage with a hollow retro-reflector was used to manipulate the signal delay time and pair the arrival timings of the gating and probe pulses. Due to dispersion of light, the arrival times of the Raman signal at the Kerr gate medium varies across the spectral range (by *ca.* 2 ps) and, therefore, a number of time delays (surrounding the optimal arrival time) were collected for each data point. After optimization of the focal position and delay times, the Kerr gated Raman spectra were then collected, while the sample was under galvano/potentiostatic control, in 40 s accumulations with a laser power of 5 mW. The sample was raster scanned continuously throughout the repeated spectral collection (and the electrochemical cycling in parallel) to limit laser damage to the electrode sample.

Operando continuous wave (CW) Raman measurements - A second operando Li|graphite cell for the CW Raman measurements was prepared as above using the equivalent electrode and electrolyte materials and cell components. The CW Raman spectra were collected using a Renishaw In-Via Raman spectrometer coupled with a Leica inverted microscope with a 50x objective. The excitation wavelength for CW Raman measurements was 633 nm and the laser power was adjusted to *ca.* 0.4 mW. The CW Raman laser spot was focused, *via* the CaF_2 window and hole in the Cu current collector, on an electrode region that resulted in minimal signals from the electrolyte. The cell was discharged from OCP (*ca.* 3 V *vs.* Li^+/Li) at C/7 to a voltage limit of 10 mV *vs.* Li^+/Li. As for the operando Kerr gated Raman measurements, the voltage was then held at 10 mV *vs.* Li^+/Li until the current decayed to C/40 to allow complete lithiation of the graphite

working electrode. CW Raman spectra were collected in parallel with the electrochemical cycling of the cell.

Supplementary Data

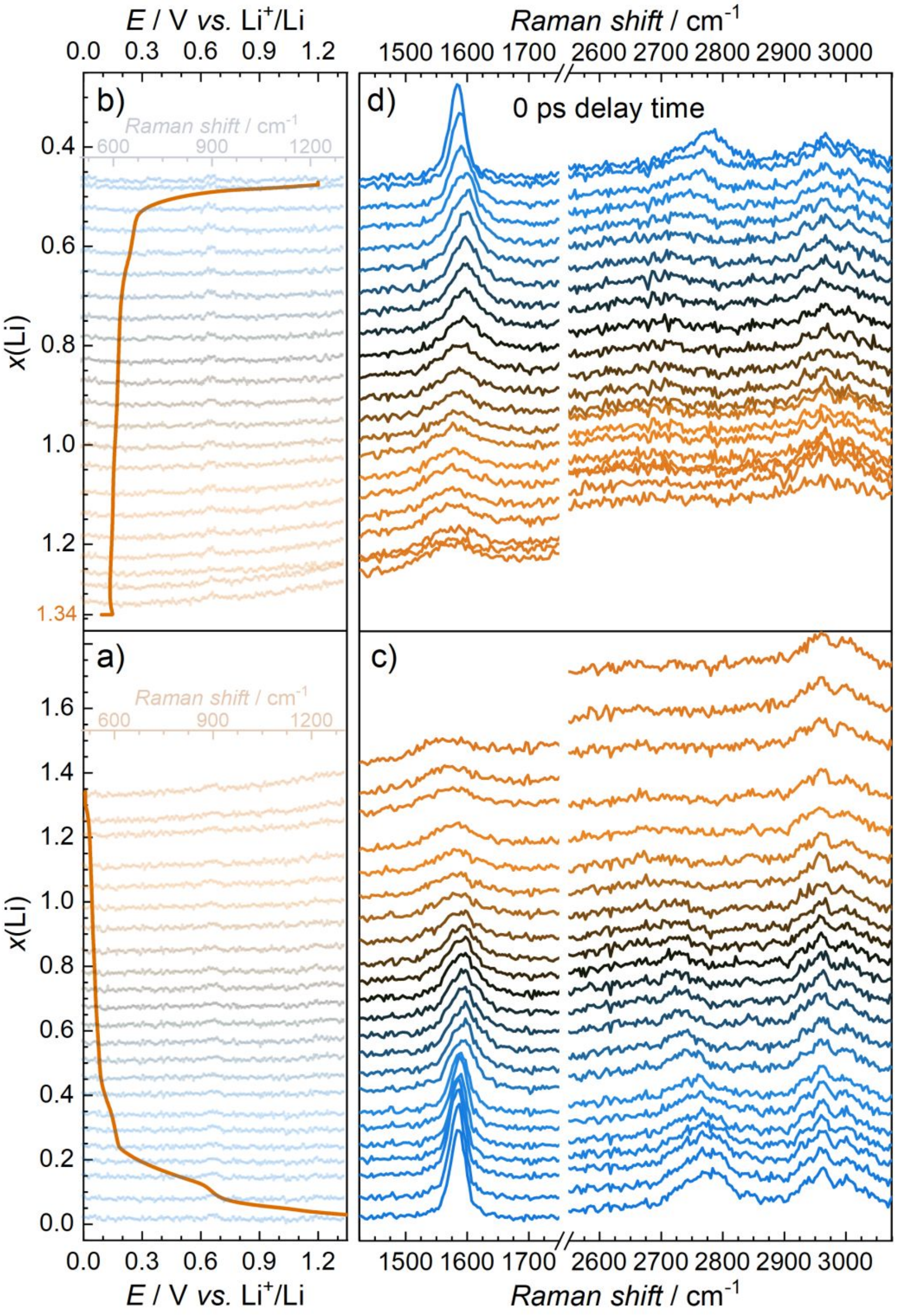

Figure S 2. Operando Kerr gated Raman spectroscopy of the lithium/graphite half-cell at the 0 ps delay time. (a, b) Voltage profiles of a graphite electrode under galvanostatic control as shown in Figure 2 in the main text; (c, d) *operando* Kerr gated Raman spectra collected at 0 ps delay times showing the primary G and 2D bands of graphite at 1586 and 2780 cm-1, respectively (electrolyte bands at *ca.* 2980 cm^{-1}). The spectra are stack plotted as a function of the depth of lithiation (x(Li)) and are not baseline subtracted. The faded spectra in panels a) and b) show the low wavenumber (500-1300 cm^{-1}) spectra regions to highlight the intersect with the depth of lithiation (accounting for the large emission baseline at higher wavenumbers for high depths of lithiation).

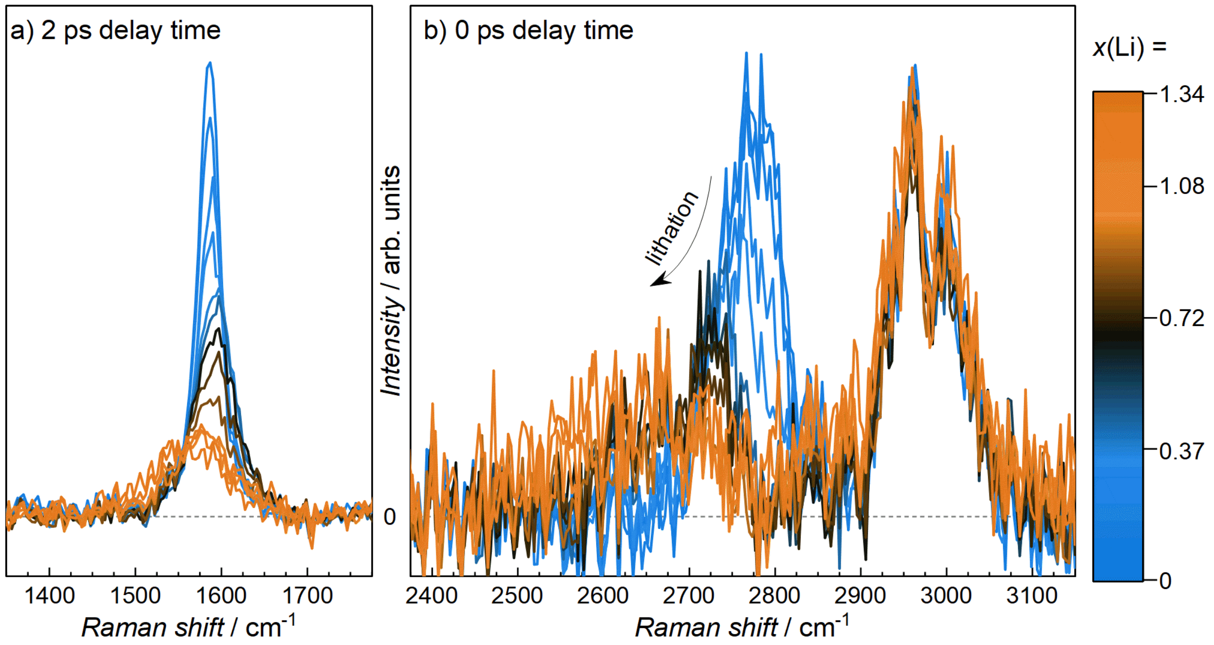

Figure S 3. Baseline subtracted, operando Kerr gated Raman spectra of the (a) G-band (2ps delay time) and (b) 2D band (0 ps delay time) regions of the graphitic electrode during the lithiation stage. The colormap corresponds to the state of lithiation (x(Li)) of the graphite electrode for each spectral collection. The zero-intensity baseline (grey dashed line) is presented but the absolute intensity is scaled independently for each panel (due to the significantly lower intensity of the 2D band feature).

The trends in the G-band features and the retention of the Raman scattering intensity at high depths of lithiation, as discussed in depth in the main text and below, are clearly represented in the spectra in Figure S3a. Figure S3b also shows the clear red-shift and loss of Raman scattering intensity of the 2D band feature as a function of depth of lithiation. A peak shift from around 2780 cm^{-1} to 2710 cm^{-1} is clearly observed as the depth of lithiation reaches x(Li) ≈ 0.65 (where the cell voltage is 60-70 mV *vs.* Li^+/Li). Beyond this point, residual positive intensities can be observed shifting further to lower wavenumbers (down to 2665 cm^{-1}) all the way to complete lithiation to x(Li) ≈ 1.34 (cell voltage = 10 mV *vs.* Li^+/Li). However, particularly with the competing growth in the emission baseline that is not completely removed by the Kerr gating effect, unambiguous assignment of such features is challenging with the observed scattering intensities and signal-to-noise ratios. Further optimization of the technique towards maximizing detectable signal intensities would be important to fully assess the nature of the 2D band in stages 2 and 1.

The presented spectra in Figure S3b also shows, that within the measurement conditions reported here, the bulk electrolyte signals (2920 – 3030 cm^{-1}) remain unchanged through the electrochemical cycling experiment. The different delay time spectra are presented here for the 2 spectral regions due to the resulting difference in signal intensities (*i.e.,* high wavenumber signals, like the 2D feature, appear with greater signal intensity for the 0 ps delay time spectra, and vice versa).

Table S 1. Peak positions and full-width at half maximum (FWHM) of the G-band Raman peak of the graphitic working electrode at high depths of lithiation (x(Li) in Li_xC_6) and low cell voltages (E) versus the Li-metal counter electrode. The true state of lithiation ([x(Li)-SEI]) in Li_xC_6 is estimated by subtracting an estimation of SEI charge contributions (equating to ≈0.35) from x(Li).

E / V *vs.* Li^+/Li	x(Li)	[x(Li)-SEI]	Peak position / cm^{-1}	FWHM / cm^{-1}
0.058	0.77	0.42	1590.4	60.5
0.054	0.83	0.48	1585.6	68.4
0.049	0.90	0.55	1582.6	70.1
0.045	0.97	0.62	1581.5	81.7
0.042	1.03	0.68	1580.2	77.8
0.038	1.09	0.74	1577.5	84.1
0.032	1.19	0.84	1572.5	106.1
0.024	1.26	0.91	1566.9	119.5
0.013	1.29	0.94	1564.8	97.4
0.010	1.32	0.97	1563.8	108.6

The peak positions for the G-band at the high states of charge for the graphite electrode show clear red shift trends that (as discussed in the main text and in the below figure) can be fitted to extract information on the state of lithiation of graphite as a function of the Raman shift. Likewise, the FWHM for the studied peaks follows a general trend towards increased broadening (larger FWHM) as a function of the depth of lithiation. With improved spectral resolution and intensity, such an observed trend could be coupled with the assigned peak positions to improve the capabilities/reliabilities for accurately probing the true state of charge/lithiation in the Li_xC_6 negative electrode.

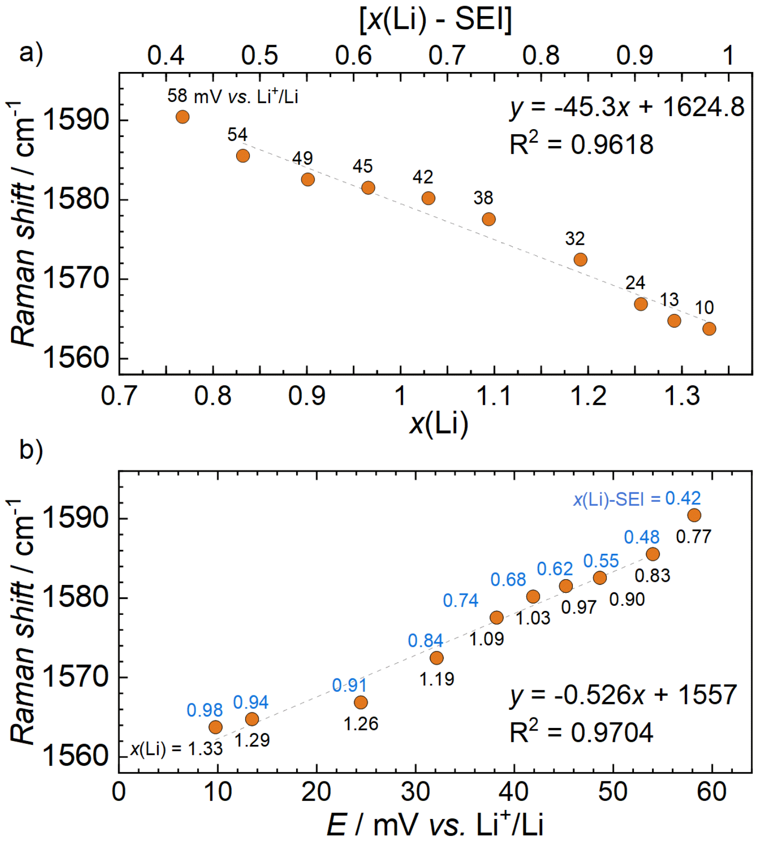

Figure S 4. A reproduction of Figure 4 in the main text also showing the linear fitting (grey dashed line) of the peak positions of the fitted G-band peak at high depths of lithiation as a function of a) the depth of lithiation (x(Li)) and b) the cell voltage *(E)*. The top x-axis of the represents an estimation of the state of lithiation in Li_xC_6 (where $0<x<1$) by subtracting charge contributions from the irreversible SEI formation (estimated as $0.35 \cdot x$(Li)). The data labels in a) shows the associated cell voltage (in mV *vs*. Li^+/Li). The top (blue) and bottom (black) data labels in b) show the associated state of lithiation with and without subtraction of the SEI contribution, respectively.

Herein, the highest voltage (lowest depth of lithiation) peak at *ca*. 1590 cm^{-1} was excluded from the linear fit of the data since, at this stage of the intercalation process, it is difficult with the low resolution of the Kerr gated Raman spectra to be confident in the loss of the distinct interior E_{2g2}(i) and bounding E_{2g2}(b) modes that constitute this peak in Stage 4-3 GICs.

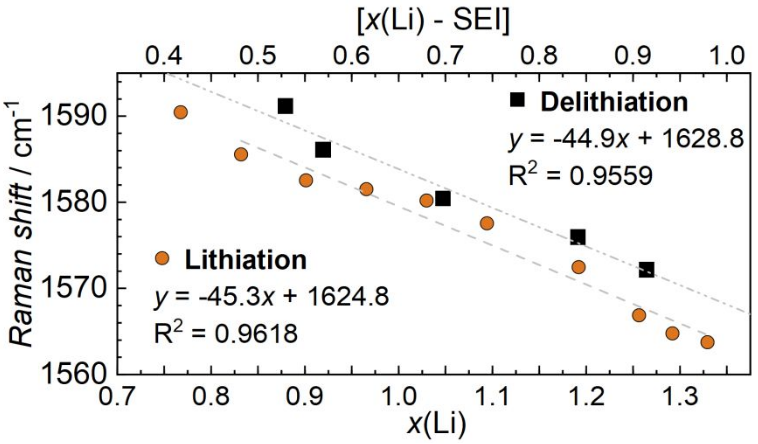

Figure S5. Comparison of the fitted peak positions and linear fitting for the shifts in the graphitic G-band during operando electrochemical lithiation (orange circles) and delithiation (black squares) for the high depths of lithiation.

Herein, the fitted positions for the observed G-band shift along the same slope for both the lithiation and the delithiation steps. This shows the marginally higher current applied on the charging (delithiation) of the Li|graphite cell has limited effect on the measured changes in the graphite electrode.

References

1. Cabo-Fernandez, L.; Neale, A. R.; Braga, F.; Sazanovich, I. V.; Kostecki, R.; Hardwick, L. J., Kerr gated Raman spectroscopy of $LiPF_6$ salt and $LiPF_6$-based organic carbonate electrolyte for Li-ion batteries. *Phys. Chem. Chem. Phys.* **2019,** *21* (43), 23833-23842.

2. Greetham, G. M.; Burgos, P.; Cao, Q.; Clark, I. P.; Codd, P. S.; Farrow, R. C.; George, M. W.; Kogimtzis, M.; Matousek, P.; Parker, A. W.; Pollard, M. R.; Robinson, D. A.; Xin, Z.-J.; Towrie, M., Ultra: A Unique Instrument for Time-Resolved Spectroscopy. *Appl. Spectrosc.* **2010,** *64* (12), 1311-1319.
